# Mitochondrial fusion and fission proteins as novel therapeutic targets for treating cardiovascular disease

**DOI:** 10.1016/j.ejphar.2015.04.056

**Published:** 2015-09-15

**Authors:** Sang-Bing Ong, Siavash Beikoghli Kalkhoran, Hector A. Cabrera-Fuentes, Derek J. Hausenloy

**Affiliations:** a Cardiovascular and Metabolic Disorders Program, Duke-National University of Singapore, Singapore; b National Heart Research Institute Singapore, National Heart Centre Singapore, Singapore; c Department of Clinical Sciences, Faculty of Biosciences and Medical Engineering, Universiti Teknologi Malaysia, Johor Bahru, Malaysia; d The Hatter Cardiovascular Institute, Institute of Cardiovascular Science, University College London, UK; e The National Institute of Health Research University College London Hospitals Biomedical Research Centre, UK; f Institute of Biochemistry, Medical School, Justus-Liebig University, Giessen, Germany; g Department of Microbiology, Kazan Federal University, Kazan, Russian Federation

**Keywords:** Mitochondrial fusion, Mitochondrial fission, MFN1, MFN2, Drp1, OPA1

## Abstract

The past decade has witnessed a number of exciting developments in the field of mitochondrial dynamics – a phenomenon in which changes in mitochondrial shape and movement impact on cellular physiology and pathology. By undergoing fusion and fission, mitochondria are able to change their morphology between elongated interconnected networks and discrete fragmented structures, respectively. The cardiac mitochondria, in particular, have garnered much interest due to their unique spatial arrangement in the adult cardiomyocyte, and the multiple roles they play in cell death and survival. In this article, we review the role of the mitochondrial fusion and fission proteins as novel therapeutic targets for treating cardiovascular disease.

## 1 Introduction

**Fig. 1:**
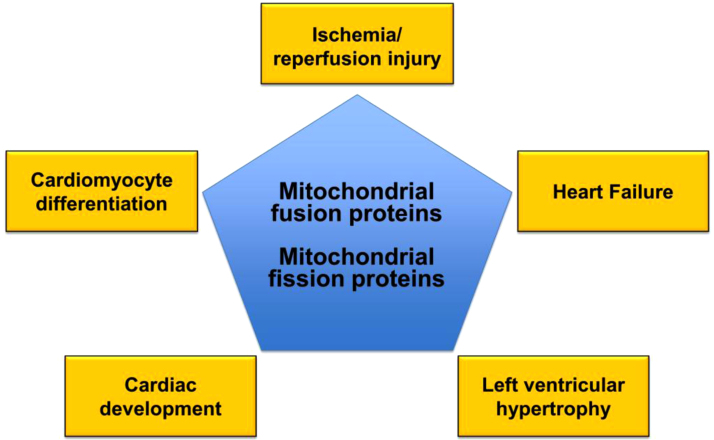
This scheme provides an overview of the role of the mitochondrial fusion and fission proteins as potential therapeutic targets for treating cardiovascular disease.

In the heart, the mitochondria occupy nearly one third the volume of a cardiomyocyte – they sustain the energy required for normal cardiac contractile function by producing up to 30 kg of adenosine triphosphate (ATP) per day. However, the role of cardiac mitochondria extends far beyond that of being merely the ‘powerhouse’ of the cell. The past decade has witnessed a number of developments in the field of mitochondrial dynamics – a phenomenon in which changes in mitochondrial shape and movement have been demonstrated to impact on cellular physiology and pathology. Mitochondria are dynamic organelles which are able to change their shape by undergoing fusion to generate elongated interconnected mitochondrial networks, and fission to produce discrete fragmented mitochondria. These processes are under the regulation of the mitochondrial fusion and fission proteins, respectively, and are essential for maintaining a healthy mitochondrial network. The cardiac mitochondria, in particular, have garnered much interest due to their unique spatial arrangement in the adult heart, and the multiple roles they play in cell death and survival. It is known that the actions of the mitochondrial fusion and fission proteins extend beyond those of mediating changes in mitochondrial shape – in this regard these pleiotropic roles may impact on their effects in the heart and the vasculature (see Fig. 1). In this article, we review the potential role for the mitochondrial fusion and fission proteins as novel targets for treating cardiovascular disease.

## 2 Mitochondrial fusion

The fusion of two adjacent mitochondria allows the mixing of intra-mitochondrial proteins and the replacement of damaged mitochondrial DNA (mtDNA) (Legros et al., 2002). In the outer mitochondrial membrane (OMM), Mitofusins 1 (MFN1) and 2 (MFN2) mediate the fusion of the OMM, whereas in the inner mitochondrial membrane (IMM), Optic Atrophy 1 (OPA1), governs the fusion of the IMM (reviewed in Youle and van der Bliek (2012)). The mitochondrial fusion proteins contain a GTPase domain, a transmembrane domain, and a coiled-coil domain. The proteins are anchored to the mitochondrial membrane by the transmembrane domain and it is the coiled-coil domains facing the cytosol that mediate the formation of homotypic (MFN1–MFN1, MFN2–MFN2 and OPA1–OPA1) or heterotypic (MFN1–MFN2) physical connections (Chen et al., 2003). These links bring adjacent mitochondria together and initiate the fusion of the OMM (Koshiba et al., 2004, Chan, 2006), while the formation of OPA1–OPA1 homotypic complexes fuse the IMM.

### 2.1 The Mitofusins (MFN1 and MFN2)

The main role of the Mitofusins is to mediate the fusion of the OMM of adjacent mitochondria. The GTPase activity of the Mitofusins is regulated by guanine nucleotide binding protein-β subunit 2 (Gβ2) (Zhang et al., 2010), and in this regard, MFN1 has been reported to have a higher GTPase activity as compared to that of MFN2 (Ishihara et al., 2004). The pro-fusion effects of MFN1 can be promoted by binding of Gβ2 to MFN1 which decreases its motility and facilitates its clustering at specific foci on the OMM (Zhang et al., 2010). MFN2, however, is regulated by expression levels rather than other post-translational modifications. One such scenario is during mitochondrial biogenesis where PGC1-α and PGC1-β up-regulate MFN2 expression to promote mitochondrial fusion (Zorzano et al., 2009, Liesa et al., 2008). Nevertheless, the ubiquitination of MFN1 and MFN2 promotes the degradation of these proteins allowing unopposed mitochondrial fission during the selective removal of dysfunctional mitochondria by mitophagy (Tanaka et al., 2010; Gegg et al., 2010).

#### 2.1.1 Pleiotropic non-fusion roles of Mitofusins

The role of the Mitofusins extends beyond that of mediating mitochondrial fusion of the OMM – this makes the investigation of these proteins in the adult heart quite challenging. Pleiotropic non-fusion actions of the Mitofusins have been mainly described for MFN2 although emerging studies suggest that MFN1 may also have non-fusion effects.

##### 2.1.1.1 MFN2 as a tethering protein

MFN2 has been shown to tether the endoplasmic reticulum (ER) to mitochondria thereby allowing the formation of subcellular calcium (Ca^2+^) domains in close proximity to the mitochondrial calcium uniporter, and facilitating the transfer of Ca^2+^signalling from the ER to mitochondria (De Brito and Scorrano, 2008). In the heart, efficient Ca^2+^ transfer from the sarcoplasmic reticulum (SR) to mitochondria is essential to tightly couple the energy requirements for cardiac contractility to mitochondrial energy production (Y. Chen et al., 2012). It has been demonstrated that in mice lacking cardiac-specific MFN2, the SR-mitochondrial tethering is disrupted resulting in impaired Ca^2+^ signalling, diminished mitochondrial respiratory function and a deterioration in left ventricular (LV) systolic function.

##### 2.1.1.2 MFN2 and apoptotic cell death

The interactions between pro-apoptotic proteins such as BAX and BAK which translocate to and permeate the OMM, and the mitochondrial fusion and fission proteins remain to be fully elucidated (Youle and Strasser, 2008). Both BAX and BAK have been demonstrated to co-localise with MFN2 in the OMM (Karbowski et al., 2002, Neuspiel et al., 2005). The binding of BAX to MFN2 has been shown to inhibit its pro-fusion function (Neuspiel et al., 2005). MFN2 may also promote mitochondrial pore formation and decrease stability of the mitochondrial membrane thereby facilitating Drp1-mediated mitochondrial fission (Papanicolaou, Phillippo, et al., 2012). The potential role of non-oligomerised or monomeric BAX and BAK in mitochondrial fusion has recently been evaluated in the context of mitochondrial permeability transition pore (MPTP) opening and necrotic cell death (Whelan et al., 2012). Combined ablation of BAX and BAK was found to promote mitochondrial fragmentation, yet the mitochondria were still shown to be resistant to MPTP opening and necrotic cell death. Interestingly, MFN2-deficient cells (Whelan et al., 2012) also exhibited resistance against MPTP opening, supporting the observation that MFN2 may promote this process together with BAX. This notion is based on the speculation that BAX localisation to the OMM may facilitate the formation of hemifusion-related holes which can be used in the exchange of ions during MPTP opening in the presence of stress. The expression of IF1 (the inhibitor of the F1 complex of the ATP synthase) relative to the F_1_F_0_-ATP synthase prevents cell death mediated by release of cytochrome *c* (cyt*c*), elevated release of ER [Ca^2+^], recruitment of Drp1 and BAX insertion into the OMM (Faccenda et al., 2013). There appears to be significant crosstalk between apoptosis and necrosis based on the finding that cells and mitochondria lacking BAX and BAK are resistant to MPTP opening and necrosis, suggesting that BAX and BAK play distinct roles in regulating both apoptosis and necrosis (Whelan et al., 2012). This was further shown where BAX/BAK/cyclophilin D triple knockout mice did not appear to exhibit a further reduction in myocardial infarct size when compared to the BAX/BAK double knockout mice (Whelan et al., 2012). In addition, the positioning of the BH3-only proteins may also dictate whether apoptosis or necrosis ensues (Diwan et al., 2009, Chen et al., 2010).

##### 2.1.1.3 MFN2 as a mediator of mitophagy

Cardiac-specific deletion of MFN2 has been reported to impair cellular autophagy as evidenced by the accumulation of autophagosomes (Zhao et al., 2012). The actual mechanism underlying the role of MFN2 in the autophagic response was not elucidated, although MFN2 was shown to associate with RAB7, an autophagosome maturation-related protein (Zhao et al., 2012). A recent study has delineated the role of MFN2 in mitophagy. Damaged mitochondria lose their mitochondrial membrane potential which then induces the translocation of PINK1 to the OMM (Leboucher et al., 2012), where it phosphorylates MFN2 at Thr-111 and Ser-442, thereby allowing the binding of Parkin, which then ubiquitinates MFN2 (Chen and Dorn, 2013). The ubiquitination of MFN2 inhibits its pro-fusion activity allowing mitochondrial fission, while recruiting p62 to facilitate selective removal of the damaged mitochondrion by mitophagy (Chen and Dorn, 2013; Gegg et al., 2010; Tanaka et al., 2010; Glauser et al., 2011).

### 2.2 OPA1

The IMM fusion protein, OPA1, mediates fusion of the IMM of two adjacent mitochondria. OPA1 function is determined by alternative splicing and post-translational modification, making its regulation somewhat complex, and only a simplified overview is presented here (for a more detailed review please see Burke et al. (2015)).

#### 2.2.1 Pleiotropic non-fusion roles of OPA1

The IMM mitochondrial fusion protein, OPA1, has been reported to display a number of non-fusion roles which have been shown to impact on cristae morphology and mitochondrial respiratory efficiency.

##### 2.2.1.1 OPA1 and mitophagy

A recent study by Sadoshima's group has demonstrated that Drp1 down-regulation elongates the mitochondria but inhibits mitophagy and causes mitochondrial dysfunction, thereby promoting LV dysfunction and increased susceptibility to I/R injury (Ikeda et al., 2015). Under conditions of stress, Parkin is recruited to the linear ubiquitin assembly complex and increases linear ubiquitination of NF-κB essential modulator (NEMO), which is essential for canonical NF-κB signalling. The NF-κB-responsive promoter elements then signals for up-regulation of OPA1 to maintain mitochondrial integrity and protect from cell death. The lack of mitophagy, however, did not hamper the Parkin-induced protection (Müller-Rischart et al., 2013).

##### 2.2.1.2 Cristae remodelling and mitochondrial apoptosis

By regulating mitochondrial cristae morphology, cyt*c* distribution, and apoptotic cell death, OPA1 has been shown to exert a strong anti-apoptotic effect. It is well-established that cristae remodelling (cristae fusion and widening of the cristae junction) by tBID is required for the redistribution of cyt*c* from the intra-cristal space into the intermembrane space (IMS) and the initiation of apoptosis (Scorrano et al., 2002; Kim et al., 2004; Frezza et al., 2006; Epand et al., 2002). By ‘stapling’ these cristae junctions closed, OPA1 has been shown to prevent the redistribution of cytochrome *c*, thereby preventing cytochrome *c* release and inhibiting apoptotic cell death (Frezza et al., 2006). These findings implicate OPA1 as a critical regulator of apoptotic cell death and therefore a therapeutic target for protecting against apoptosis.

##### 2.2.1.3 Cristae remodelling and mitochondrial respiratory efficiency

The respiratory complexes of the electron transport chain (ETC) are assembled into respiratory chain supercomplexes (RCS), the arrangement of which facilitates the transfer of electrons between the respiratory complexes, thereby improving mitochondrial respiratory efficiency (reviewed in Saraste (1999) and Schäfer et al. (2006)). The regulation of cristae morphology by OPA1 has been recently shown to impact on the formation of RCS and mitochondrial energy production. Using genetic manipulation of OPA1, Cogliati et al. (2013) have demonstrated that the stability and assembly of RCS, mitochondrial respiratory efficiency, and mitochondria-dependent cell growth were critically dependent on cristae morphology. These findings implicate OPA1 as a critical regulator of mitochondrial respiration and therefore a therapeutic target for modulating mitochondrial energy production.

## 3 Mitochondrial fission

Mitochondrial fission ensures equal division of mitochondrial numbers during cell division and mediates the selective removal of damaged mitochondria by the process of mitophagy. The process of mitochondrial fission is mediated by Drp1 which translocates from the cytosol to the OMM where it interacts with other proteins of the fission machinery including human fission protein-1 (hFis1), mitochondrial fission factor (Mff), and mitochondrial dynamics proteins of 49 (MiD49) and 51 kDa (MiD51), although the actual interplay between these proteins remains unclear (reviewed in Otera et al. (2013) and Elgass et al. (2013)). At the OMM, Drp1 then oligomerises forming a spiral which encircles the mitochondrion and mediates the scission of the latter. It appears that Drp-1 mediated mitochondrial fission is initiated by early constriction of mitochondria after making contact with the endoplasmic reticulum (ER) (Friedman et al., 2011), through the association of the ER-associated inverted formin 2 (INF2, a formin that accelerates both actin polymerisation and depolymerisation) and the actin component of the cytoskeleton (Korobova et al., 2013, De Vos et al., 2005). It has been suggested that the ER encircles mitochondria at sites of fission, and ER-associated INF2 then stimulates actin polymerisation, providing the force required for partial constriction of the mitochondria, thereby facilitating the translocation of Drp1 to these pre-constriction contact sites in the OMM. The actual mechanism through which Drp1 localises to these pre-constricted ER-contact sites on the OMM, and the roles which hFis1, Mff and MiD49/51 play in this process remains to be determined.

The translocation of Drp1 from the cytosol to the mitochondria is regulated by a number of different post-translational modifications including SUMOylation (Figueroa-Romero et al., 2009), phosphorylation (Cribbs and Strack, 2007, Cho et al., 2010, Chang and Blackstone, 2007), ubiquitination (Nakamura et al., 2006), S-nitrosylation (D.-H. Cho et al., 2009), and O-GlcNAcylation (Gawlowski et al., 2012). The phosphorylation of Ser-637 by protein kinase A (PKA) (Cribbs and Strack, 2007; Chang and Blackstone, 2007), Ca^2+^/calmodulin-dependent protein kinase (CaM Kinase) (Han et al., 2008), and Proto-oncogene serine/threonine-protein kinase Pim-1 (Pim1) (Din et al., 2013) has been shown to prevent the mitochondrial translocation of Drp1. In contrast, the phosphorylation of Ser-616 by Cdk1/cyclin B (a key mitotic kinase) promotes mitochondrial fragmentation by Drp1 during mitosis (Taguchi et al., 2007, Marsboom et al., 2012). Under conditions of high cytosolic Ca^2+^, dephosphorylation of Drp1 at Ser-637 by calcineurin induces mitochondrial fission (Cribbs and Strack, 2007, Cereghetti, 2008, Cho et al., 2010, Sandebring et al., 2009, Estaquier and Arnoult, 2007). In hyperglycaemic conditions, O-GlcNAcylation of OPA1 (Makino et al., 2011) and Drp1 (Gawlowski et al., 2012) causes dephosphorylation of Ser-637 and the translocation of Drp1 to the OMM.

### 3.1 Drp1 and cell death

In addition to co-localisation with BAX at the OMM, Drp1 has also been reported to be recruited by BAX in response to apoptotic stimuli (Karbowski et al., 2002) and stabilised by SUMO-mediated ubiquitination (Wasiak et al., 2007). Drp1 promotes mitochondrial fragmentation, loss of MMP and release of cyt*c* when localised to the OMM. Nevertheless, Drp1 inhibition only slows down rather than fully inhibits apoptosis as other pro-apoptotic proteins may still be released from the mitochondria (Estaquier and Arnoult, 2007, Frank et al., 2001). Furthermore, there is growing evidence that apoptosis can still occur regardless of the fragmentation of the mitochondria (Parone et al., 2006, Wakabayashi et al., 2009).

In contrast to the previously held belief that necrosis is unregulated, a pathway of programmed cell necrosis has been recently described.The cytokine TNF-α activates the receptor-interacting serine–threonine kinases RIP1 and RIP3 which interact with the mixed lineage kinase domain like protein (MLKL) resulting in ROS generation, Ca^2+^overload, and the opening of the MPTP (Vanlangenakker et al., 2008; Y.S. Cho et al., 2009; Chen et al., 2013). RIP3 forms a complex with the mitochondrial protein phosphatase PGAM5, which recruits Drp1 to the OMM by dephosphorylating its Ser-637 site to cause mitochondrial fragmentation (Wang et al., 2012), although Drp1 was deemed to be unnecessary in necroptosis induced by RIPK3 in more recent findings (Moujalled et al., 2014, Remijsen et al., 2014). These findings implicate a role for Drp1 in mediating programmed cell death necrosis. Caspase-8 also suppresses the RIP3–RIP1 kinase complex-dependent necroptosis by proteolytic cleavage (He et al., 2009). Caspase-8 deficient embryos die between embryonic days 10.5 and 11.5 due to the activation of RIP3 (He et al., 2009). Double KO of RIP3 and caspase-8 avoided mortality and allowed for maturation into adult (Kaiser et al., 2011). Pharmacological inhibition of this pathway, using Necrostatin-1 to target RIP1, has been reported to limit MI size and prevent post-LV remodelling in cell lines as well as animal models of acute IRI (Oerlemans et al., 2012, Lim et al., 2007; L. Zhang et al., 2013). Repression of RIP3 or MLKL blocks necroptosis but activates RIP1-dependent apoptosis although the possibility of this being due to different species was raised (Remijsen et al., 2014). In addition, the different identities of cell death initiators may dictate the pathway of necroptosis (Obitsu et al., 2013). The signalling events underlying necroptosis have been shown to involve Akt/mTOR as well in the neuron model where cell death was preceded by RIPK1-RIPK3-pAkt assembly (Liu et al., 2014).

## 4 Mitochondrial fusion and fission proteins and the adult heart

In the adult heart where the unique spatial arrangement of mitochondria restricts their mobility and distributes them into 3 distinct subpopulations (subsarcolemmal, interfibrillar, and perinuclear), the relevance of mitochondrial dynamics is not easily apparent. Although relatively immobile, experimental studies have demonstrated that cardiac mitochondria do form an interconnected network, and both fusion and fission events do occur thus allowing for the exchange of mitochondrial contents and the selective removal of damaged mitochondria within the cardiomyocyte, albeit at a much slower rate. By tracking the movement of photo-activatable green fluorescentprotein (PAGFP) across the cardiomyocyte over several hours, Huang et al. (2013) were able to demonstrate that cardiac mitochondria formed an interconnected network which allowed for the exchange of mitochondrial content.

### 4.1 Cardiac development and maturation

Genetic ablation of both MFN1 and MFN2 causes death in utero during the mid-gestation period (Chen et al., 2003) whereas cardiac-specific ablation of both Mitofusins in the embryo was shown to be lethal at day E9.5–10.5 (Chen et al., 2011). Together, these findings imply a role for the Mitofusins in cardiac development. The complete ablation of OPA1 causes in utero death, thus underscoring the importance of this protein in embryonic development and maintenance of mitochondrial integrity (Davies et al., 2007). Finally, the Drp1 knockout mouse is embryonically lethal at day E12.5 confirming an essential role for this mitochondrial fission protein in embryonic development (Manczak et al., 2012).

In the first few days of post-natal growth, major changes in cardiac metabolism and intracellular architecture occur – these are required to support the increased energy required for cardiac growth and maturation. In order to support a change in mitochondrial respiration from glycolysis to oxidative phosphorylation, there is a shift in mitochondrial morphology from a fragmented phenotype to elongated mitochondria aligned with the myofibrils. This change in mitochondrial morphology is reliant on the increased levels of Mitofusins present at this time (Papanicolaou, Kikuchi, et al., 2012). Furthermore, transgenic mice (loxP/Myh6-Cre) deficient in cardiac-specific MFN1 and MFN2 from the late embryonic period displayed severe mitochondrial dysfunction at 7 days (abnormal mitochondrial structure, down-regulated mitochondrial biogenesis genes, reduced mitochondrial DNA), developed cardiomyopathy, and all died before 14 days old (Papanicolaou, Kikuchi, et al., 2012). The shift towards a more elongated phenotype also depends on a reduction in cardiac hypoxia-inducible factor (HIF) signalling, which mediates an increase in fusion protein expression from E16.5 in a stepwise manner through to P10.5 with the largest increase taking place between P0.5 and P2.5 in MFN1 and MFN2 and between P2.5 and P10.5 in OPA1 while no changes were detectable in fission protein Drp1 and Fis1 levels over this time period (Neary et al., 2014). Postnatal growth of the heart also witnesses a role for PGC-lα in stimulating the transcription of MFN1 gene by co-activating the orphan nuclear receptor ERRα. Nonetheless, PGC-1 co-activators are dispensable for maintenance of mitochondria in the adult heart, but required for a high-level expression of nuclear- and mitochondrial-encoded genes involved in mitochondrial energy transduction and OXPHOS pathways, and for full respiratory capacity (Martin et al., 2014).

Another critical change which occurs at the time of birth is closure of the ductus arteriosus (DA) in response to the increase in oxygen thereby diverting blood from the right ventricle into the pulmonary circulation. Failure of the DA to close can result in pulmonary congestion and failure to thrive. The presence of oxygen in this setting rapidly induces phosphorylation of Drp1 at Ser-616 in less than 5 min to promote fragmentation of mitochondria in DA smooth muscle cells. The fragmentation of mitochondria induced production of signalling ROS which activated closure of the DA via inhibition of oxygen-sensitive potassium channels. L-type channel activation increased intracellular calcium and subsequent vasoconstriction of the DA (Hong et al., 2013).

### 4.2 The Mitofusins and the adult heart

Genetic ablation of both MFN1 and MFN2 causes death in utero during the mid-gestation period (Chen et al., 2003) whereas cardiac-specific ablation of both Mitofusins in the embryo has been shown to belethal at day E9.5–10.5 (Chen et al., 2011). Together, these findings imply a role for the Mitofusins in cardiac development. Conditional ablation of cardiomyocyte-specific MFN2 (α-MHC-*Cre*) in the adult murine heart causes mitochondria to become pleiomorphic and slightly enlarged while the hearts displayed modest LV hypertrophy and a mild deterioration in LV systolic function (Chen et al., 2012, Papanicolaou et al., 2011). In contrast, the genetic ablation of cardiac-specific MFN1 in the adult heart did not appear to induce a marked cardiac phenotype (Papanicolaou, Nogh, et al., 2012). As expected, the combined ablation of both cardiac MFN1 and MFN2 in the adult murine heart resulted in mitochondrial fragmentation with disordered cristae morphology and induced a lethal cardiomyopathy after several weeks (Papanicolaou and Kikuchi, 2012, Chen et al., 2011).

### 4.3 OPA1 and the adult heart

The role for OPA1 in the adult heart has been investigated in mice partially deficient in this protein, given that the complete ablation of OPA1 causes in utero death (Davies et al., 2007). The transgenic OPA1 +/− mice has a 50% reduction in myocardial OPA1 and displays enlarged mitochondria with disrupted cristae and altered mitochondrial organisation. These mice exhibit only a mild cardiac phenotype at 3 and 6 months but start to develop heart failure at 12 months. The mutation in OPA1 causes cardiac dysfunction with a reduced cardiac output, blunted inotropic reserve and impaired pressure–volume loops (L. Chen et al., 2012b).

### 4.4 Drp1 and the adult heart

Genetic ablation of Drp1 is embryonically lethal at day E12.5 (Manczak et al., 2012). Cardiac-specific ablation of Drp1 in the adult heart has been shown to produce a cardiomyopathy due to mitochondrial dysfunction arising from an impaired mitophagic response (Ikeda et al., 2015), underscoring the importance of Drp1-mediated fission in maintaining a healthy mitochondrial network. These findings will limit inhibition of Drp1 as a therapeutic approach to the acute setting, given that chronic Drp1 inhibition is likely to be detrimental for cardiac function.

## 5 Mitochondrial fusion and fission proteins in cardiovascular disease

### 5.1 Vascular smooth muscle cell proliferation

Vascular smooth muscle cell (VSMC) proliferation and hyperplasia have been identified to be hallmark features in a variety of cardiovascular diseases including coronary atherosclerosis, hypertension and pulmonary arterial hypertension (PAH). The existence of VSMC proliferation and hyperplasia causes failure of coronary artery bypass vein grafts and restenosis following percutaneous coronary intervention. As such, novel therapeutic targets are required to inhibit VSMC proliferation in these settings. In this regard, the mitochondrial fusion and fission proteins may provide novel targets for preventing this pathological process.

#### 5.1.1 MFN2 and VSMC proliferation

MFN2 was originally identified as a novel hyperplasia suppressor gene (HSG), capable of inhibiting VSMC proliferation in a variety of vasculo-proliferative conditions (Chen et al., 2004). The overexpression of MFN2 was shown to inhibit VSMC proliferation in an experimental animal model of angioplasty balloon-induced neointimal injury, oxidised LDL and subsequent atheroma formation and carotid artery restenosis. The anti-proliferative effect of MFN2 was found to be due to PKA-induced phosphorylation of MFN2 at Ser442 (Zhou et al., 2010). Down-regulation of MFN2 was shown to enhance VSMC proliferation, a finding which was accompanied by an increase in fatty acid oxidation and decrease in glucose oxidation. These results suggest that changes in mitochondrial morphology and bioenergetics may underlie the hyperproliferative features of the VSMC in this setting.

#### 5.1.2 Drp1 and VSMC proliferation

The role of the mitochondrial fission protein Drp1 in proliferation of VSMCs has been studied by Chalmers et al. (2012) who found that in native non-proliferative quiescent VSMCs mitochondria were fairly static mainly ovoid in shape whereas during proliferation, the mitochondria became more mobile and displayed varying shapes. Following angiotensin II (Ang II) stimulation, activated Drp1 interacted with PKC-δ and then activated MEK1/2–ERK1/2 signalling cascade and MMP2 (Lim et al., 2015). Treatment of VSMCs with the Drp1 inhibitor, *mdivi-1*, impaired the proliferative response, suggesting that mitochondrial fission may be required for VSMC proliferation (Lim et al., 2015). The requirement for mitochondrial fission in VSMC proliferation has been recently explored in the setting of PAH.

### 5.2 Pulmonary arterial hypertension

Pulmonary arterial hypertension (PAH) is defined as a condition where the pulmonary arteries become obstructed, resulting in right ventricular hypertrophy and heart failure. In this regard, recent experimental data has implicated the mitochondrial fusion and fission proteins as potential novel therapeutic targets for treating PAH (see Table 1).

#### 5.2.1 Mitochondrial fusion proteins and PAH

Similarly, both MFN2 and PGC-1α were down-regulated in pulmonary arterial smooth muscle cells (PASMC) in two different experimental models of PAH, and in patients with PAH while MFN2 reversed this phenotype. Together, these findings implicate the mitochondrial-shaping proteins as novel therapeutic targets for PAH (Marsboom et al., 2012). Although preventing PAH by using *mdivi-1* is beneficial short-term, the feasibility remains to be determined as chronic inhibition of mitochondrial fission may be detrimental.

#### 5.2.2 Mitochondrial fission proteins and PAH

Hyperproliferation of the PASMC is an essential part of the pathophysiology underlying PAH. It has been demonstrated that the ability of mitochondria to divide is central to equal re-distribution of mitochondria during cell proliferation. The fragmentation of the mitochondria was due to upregulation of Drp1 and downregulation of MFN2, coupled with Cyclin B1/CDK1 phosphorylation of Drp1 at Ser616. Interestingly, treatment with the small molecule Drp1 inhibitor, *mdivi-1*, was shown to prevent the progression of PAH in three different experimental models of PAH (Marsboom et al., 2012).

### 5.3 Heart failure

The development of heart failure has been documented to involve deficiencies in either the mitochondrial fusion or fission proteins (see Table 1). Nevertheless, whether these changes in mitochondrial-shaping proteins directly cause heart failure or merely constitute a consequence of the alteration of mitochondrial biogenesis remains to be determined (Garnier et al., 2005; Ventura-Clapier et al., 2011).

#### 5.3.1 Mitochondrial fusion proteins and heart failure

Deficiencies in cardiac MFN1 and MFN2 have been shown to result in a mild cardiomyopathy (Chen et al., 2012, Papanicolaou et al., 2011). In another study, the genetic ablation (Mlc2v-Cre) of MFN2 in the adult murine heart produced no obvious cardiac phenotype in mice up to 4 months of age (Zhao et al., 2012). It was only at 17 months of age that the MFN2-KO mice displayed increased sensitivity to acute IRI, and developed late-onset LV dysfunction (Zhao et al., 2012). These findings were associated with a major metabolic disturbance with impaired autophagy, defective lipid metabolism, and decreased mitochondrial respiration (primarily at complex III) (Zhao et al., 2012). The explanation for the observed differences in effects of MFN2 ablation in the adult heart is not clear. Whether the difference in response to MFN2 ablation can be attributed to the use of different cardiac-specific promoters is not known.

Using a post-MI rat heart failure model and human dilated and ischaemic cardiomyopathy tissue samples, the fragmentation of the mitochondria due to decreased myocardial levels of OPA1 was demonstrated (Chen et al., 2009). Partial deficiency in OPA1 also reduced mitochondrial DNA copy number and decreased expression of nuclear antioxidant genes at 3–4 months (L. Chen et al., 2012). Nevertheless, baseline cardiac function was found to be normal in these OPA1-deficient mice, although cardiomyopathy associated with mitochondrial fragmentation and impaired mitochondrial function developed at 12 months of age (L. Chen et al., 2012). The reason for the decline of OPA1 levels in heart failure requires further investigation. Similarly during initial compensatory cardiac hypertrophy, an increase in OPA1 and decrease in Drp1 occur in concert with decreased Parkin and Sirt1/AMPK-PGC-1α signalling, signifying a compromised mitochondrial remodelling system (Tang et al., 2014).

The silencing of MARF (mitochondrial assembly regulatory factor – ortholog of mammalian, Mitofusin) and OPA1 in the *Drosophila* fly heart tube causes dilated cardiomyopathy, which could be rescued by over-expressing either of the human Mitofusins (MFN1 or MFN2) or superoxide dismutase 1, implicating impaired mitochondrial fusion and oxidative stress in the pathogenesis of the heart failure (Dorn, 2011). Fragmentation of the mitochondria and impairment of mitochondrial respiration resulting from conditional cardiac-specific combined ablation of MFN1 and MFN2 in the adult murine heart have been reported to result in a severe lethal dilated cardiomyopathy after 6–8 weeks, implicating a role for the Mitofusins in maintaining normal mitochondrial function in the adult heart (Chen et al., 2011). A decrease in MFN2, an increase in Fis1, and no change in OPA1 expression were also detected in rat hearts 12–18 weeks after myocardial infarction (Javadov et al., 2011). The ablation of MFN2 in hearts also leads to impaired Parkin-mediated mitophagy causing an accumulation of damaged ROS-producing organelles and progressive heart failure. An optimally suppressed mitochondrial ROS prevented mitochondrial depolarisation, respiratory impairment, and structural degeneration in MFN2-null hearts. Super-suppressed mitochondrial ROS which was associated with impaired secondary autophagic pathways, however, failed to improve mitochondrial health, suggesting the importance of mitochondrial ROS alongside mitochondrial dynamics in mediating mitophagy and minimise cardiac failure (Song et al., 2014). Parkin deficiency and resulting mitophagic disruption causes an accumulation of enlarged hollow donut mitochondria with dilated cardiomyopathy (Bhandari et al., 2014). Suppressing mitochondrial fusion completely prevented the cardiomyopathy and corrected mitochondrial dysfunction, albeit mitochondrial dysmorphology was not normalised, demonstrating the link among improper mitochondrial fusion, defective mitophagy and organ dysfunction (Bhandari et al., 2014).

#### 5.3.2 Mitochondrial fission proteins and heart failure

Ashrafian et al. (2010) have described a novel mutation in the Drp1 gene (C452F) which gives rise to an autosomal dominant form of dilated cardiomyopathy in the python mouse. The homozygous mutation is embryonically lethal but the heterozygous form survived until adulthood and developed a severe dilated cardiomyopathy after 11 weeks, a finding which was associated with reduced content of mitochondrial respiratory enzymes and ATP (Ashrafian et al., 2010). While increased mitochondrial fission due to an up-regulation of Drp1 has been previously linked to heart failure, a decrease in SUMO attachment to Drp1 has been detected in SENP5-Tg hearts. The mitochondria of the SENP5-Tg hearts were significantly larger during the early developmental stage which would account for the pathological change observed (Kim et al., 2015). Aberrant mitophagy and elevated mitochondrial oxidative stress contribute to abnormal activation of MMP-9, leading to degradation of the gap junction protein connexin-43 (Cx-43) in the ventricular myocardium (Jansen et al., 2012; Givvimani et al., 2010). Reduced Cx-43 levels were associated with increased fibrosis and ventricular dysfunction in heart failure (Jansen et al., 2012). Treatment with *mdivi-1* normalised the decreased ratio of MFN2 to Drp1 (Gharanei et al., 2013) as well as the expression levels of MMP-9 and Cx-43, thus showing an improved cardiac function (Sharp et al., 2014; Givvimani et al., 2010; Givvimani et al., 2012).

### 5.4 Acute ischaemia–reperfusion injury

Acute IRI is derived from the opening of the MPTP at the onset of reperfusion following a sustained period of ischaemia. The susceptibility of the heart to acute IRI and its recovery is critically dependent on the function of its mitochondria. Therefore, the preservation of mitochondrial function and the prevention of MPTP opening during acute IRI are important therapeutic strategies for cardioprotection. Recent experimental data suggests that manipulating the mitochondrial fission and fusion proteins in the heart may impact on the susceptibility to acute IRI providing novel therapeutic targets for cardioprotection (see Table 1).

#### 5.4.1 Mitochondrial fission proteins and IRI

In response to acute IRI, fragmentation of the mitochondria by Drp1 was detected (Ong et al., 2010, Din et al., 2013). The inhibition of Drp1 in the HL-1 cardiac cell line using genetic or pharmacological approaches prevented the opening of the MPTP and reduced cell death. In the murine heart, pharmacological inhibition of Drp1 reduced cell death in isolated cardiomyocytes subjected to simulated IRI and reduced MI size in the adult murine heart subjected to in vivo acute IRI. Pre-treatment with *mdivi-1* in the brain significantly reduced oxidative stress, upregulated Bcl-2 expression, and downregulated Drp1, BAX, and cyt*c* expression (Wang et al., 2014; Zhao et al., 2014). In the heart, *mdivi-1* reduced mitochondrial reactive oxygen species, improved LV developed pressure, and lowered LV end diastolic pressure following IR, an effect which was also mirrored using the calcineurin inhibitor, FK506 (Sharp et al., 2014). Other studies also provided evidence that inhibition of Drp1-mediated mitochondrial fission by various upstream pathways protected the heart, e.g. over-expression of Pim1 kinase (Din et al., 2013) and usage of the non-specific dynamin inhibitor, Dynasore (Gao et al., 2013). Drp1 inhibition confers cardioprotection by reducing mitochondrial metabolism during I/R (Zepeda et al., 2014). The protective effects of inhibiting mitochondrial fission have also been detected in the kidney and the brain, suggesting that therapeutic targeting mitochondrial fission may be beneficial in other organs (N. Zhang et al., 2013). Most recently, a specific peptide inhibitor of Drp1, named P110, has been used to demonstrate that inhibiting mitochondrial fission at reperfusion can reduce myocardial infarct size and prevent adverse left ventricular remodelling post-MI in the adult rat heart (Disatnik et al., 2013) as well as in the brain (Guo et al., 2014). Futhermore, the discovery of other components of the mitochondrial fission machinery such as Mff and MiD49/51 raises the possibility of inhibiting these other proteins to mediate cardioprotection (Long et al., 2013). However, this therapeutic strategy will only be useful in protecting the heart against acute episodes of IRI as this can be achieved by transient pharmacological inhibition of mitochondrial fission. The chronic inhibition of mitochondrial fission would be detrimental to the heart and other organs as this process is critical to maintaining a healthy mitochondrial network (Ikeda et al., 2015). The opening of the MPTP following ischaemia has also been postulated to be caused by hexokinase II (HKII) dissociation from the mitochondrial contact sites at the OMM and IMM. Nevertheless, the interaction between HKII and Drp1 in maintenance of the contact sites remains to be determined (Pasdois et al., 2013; Halestrap et al., 2015). The SUMO-2/3-specific protease SENP3 is also found to be degraded during ischaemia, via a pathway involving the unfolded protein response (UPR) kinase PERK and the lysosomal enzyme cathepsin B. Ischaemia-induced cell death is suppressed as depletion of SENP3 prolongs Drp1 SUMOylation. Upon reoxygenation, recovery of levels of SENP3 allows deSUMOylation of Drp1, which facilitates Drp1 localisation at mitochondria and promotes fragmentation and cyt*c* release (Guo et al., 2013).

#### 5.4.2 Mitochondrial fusion proteins and IRI

The role of the mitochondrial fusion proteins (MFN1, MFN2 and OPA1) as targets for cardioprotection has been established, although the pleiotropic non-fusion effects need to be taken into account. Our laboratory has demonstrated that over-expressing MFN1 or MFN2 in the HL-1 cardiac cell line prevented the opening of the MPTP and reduced cell death following simulated IRI (Ong et al., 2010). Consistent with a potential protective role of the MFN2, it was demonstrated in neonatal cardiomyocytes that the genetic ablation of MFN2 increased MPTP opening susceptibility and worsened cell death; however, contrasting findings were found in MFN2-deficient adult cardiomyocytes with protection against cellular injury (Papanicolaou et al., 2011).

Although partial ablation of OPA1did not significantly alter cardiac function, the size of the mitochondria was increased with the formation of clusters of fused mitochondria and altered cristae. Interestingly, MPTP opening to calcium accumulation was less sensitive (Piquereau et al., 2012). The studies conducted so far have focused on the effects of genetically ablating the mitochondrial fusion proteins yet there is still a gap in terms of determining sensitivity to acute IRI by over-expressing the mitochondrial fusion proteins in the adult heart. In summary, the role of the mitochondrial fusion proteins in the adult heart in terms of susceptibility to acute IRI is quite complex. However, the development of small molecule inhibitors of MFN1 and MFN2 may provide a novel therapeutic strategy for cardioprotection.

Up-regulation of Hand1, a basic helix–loop–helix transcription factor highly expressed in the embryonic heart, has been demonstrated to be protective against myocardial ischaemia, forming part of a novel regulatory pathway linking cardiac oxygen levels with oxygen consumption (Breckenridge et al., 2013). During ATP-depleted ischaemia in renal proximal tubular cells, knockdown of OMA1 suppressed OPA1 proteolysis, mitochondrial fragmentation, cyt*c* release, and consequent apoptosis (Xiao et al., 2014). The pro-survival kinase, Akt as well as its pharmacological activator, EPO has also been postulated to confer cardioprotection by means of elongating the mitochondria via modulation of MFN1 (Ong et al., 2014).

### 5.5 Left ventricular hypertrophy

Hypertrophy of the left ventricle can be classified into either physiological (in response to exercise) or pathological (congenital or acquired – most often a detrimental response to an increase load). LV hypertrophy can lead to an increased risk of arrhythmias, regions of ischaemia and heart failure (Frey and Olson, 2003, Frey et al., 2004). As such novel therapeutic agents are required to prevent the progression of left ventricular hypertrophy (LVH) and reduce the onset of heart failure. Samples of RV and LV obtained from neonatal calves subjected to 2 weeks of hypobaric hypoxia showed no differences between the ventricles in terms of mitochondrial protein expression levels and mitochondrial activity (Bruns et al., 2015). Mitochondrial DNA was unchanged, as was mitochondrial content and mitochondrial dynamics. Activity of individual respiratory chain complexes was reduced (complex I) or unchanged (complex V). Key enzymes in the glycolysis pathway were upregulated in both ventricles, alongside upregulation of hypoxia inducible factor 1-α protein (Bruns et al., 2015). Whether these findings reflect a difference between developmental stages (neonatal vs adult), experimental models of inducing hypertrophy, or species differences, warrant further investigation.

#### 5.5.1 Mitochondrial fusion proteins and LV hypertrophy

MFN2 (formerly known as hyperplasia suppressor gene or HSG) has been demonstrated to inhibit proliferation of VSMC by suppressing MEK1/2–Erk1/2, a pathway which is up-regulated in LVH (Fang et al., 2007). Using different experimental models of LVH (phenylephrine induced LVH in neonatal rat cardiomyocytes, spontaneously hypertensive rats, β_2-_adrenergic transgenic mice, and pressure overload LVH by transverse aortic constriction), Fang et al. (2007) demonstrated that MFN2 expression was down-regulated and Erk1/2 up-regulated. Similarly, angiotensin-II treatment in neonatal rat cardiomyocytes decreased MFN2 expression while elevating Akt levels. Over-expressing MFN2 reversed the Ag-II-induced LVH in both neonatal cardiomyocytes and the intact rat heart (Yu et al., 2011). It is noteworthy to point that the reduction in MFN2 levels should be compared against total mitochondrial number as LVH can result in reduction of mitochondrial mass. The effect of MFN2 in cardiac hypertrophy was further elucidated using mice with cardiac-specific MFN2 knockout, where a loss of tethering to the ER may cause impaired Ca^2+^ signalling or enhanced ROS production (Chen and Dorn, 2013; Papanicolaou et al., 2011).

Partial deficiency in OPA1 also increases susceptibility to LVH and cardiac dysfunction induced by total aortic constriction (TAC) (Piquereau et al., 2012). Nevertheless, whether OPA1 over-expression could similarly reverse this phenotype remains to be investigated.

#### 5.5.2 Mitochondrial fission proteins and LV hypertrophy

Compared to the fusion proteins MFN2 and OPA1, the fission protein Drp1 was shown to be up-regulated in a cell model of phenylephrine-induced cardiomyocyte hypertrophy, suggesting a shift to mitochondrial fragmentation and enhanced levels of mitophagy may be associated with the development of LVH (Javadov et al., 2011, Givvimani et al., 2012). Inhibiting mitochondrial fragmentation with the Drp1 inhibitor *mdivi-1* enhanced the maintenance of the mitochondrial population, a release of pro-angiogenic factors (CD31 and VEGF) and a reduced collagen deposition which prevented the progression of LVH and development to heart failure induced by pressure overload TAC (Givvimani et al., 2012). These findings were also mirrored in a quantitative phosphoproteomics study by Chang et al. (2013) using myocardial samples at different time points following transverse aortic banding (TAB). Phosphorylation of DRP1 S622 and subsequent mitochondrial translocation were detected in TAB-treated mouse hearts and phenylephrine (PE)-treated rat neonatal cardiomyocytes (Chang et al., 2013). The hypertrophic response and oxygen consumption were reduced in response to treatment with *mdivi-1* (Chang et al., 2013). These findings suggest a potential therapeutic strategy in acute inhibition of mitochondrial fission proteins to salvage LV hypertrophy (see Table 1).

### 5.6 Stem cell differentiation into cardiomyocytes

The process of differentiation from cardiac stem cells to adult cardiomyocytes required changes in mitochondrial function and architecture to accommodate for the increased metabolic demands of the differentiated beating cardiomyocyte (reviewed in Rehman (2010)). Furthermore, a switch from anaerobic glycolysis to oxidative phosphorylation is crucial for the differentiation process (Chung et al., 2007). The change in mitochondrial metabolism has been reported to be associated with a change in mitochondrial morphology from a fragmented state (lacking cristae) in the ESC, to an elongated state (with well-developed cristae), closely aligned with the myofibrils of the differentiated contractile cardiomyocyte (Chung et al., 2007). The alteration in expression level of Drp1 and MFN2 mediates this change in mitochondrial morphology (Chung et al., 2007). A recent experimental study has shown that the presence of OPA1 and MFN2 is required in the development of the heart, with developmental arrest occurring at E13.5 (Kasahara et al., 2013). Differentiation from ESC to cardiomyocytes was associated with an increased expression of MFN2 and OPA1. Although knockout of MFN2 and OPA1 failed to affect mitochondrial biogenesis, the mitochondrial network failed to elongate and the cells were no longer able to differentiate into beating cardiomyocytes. The ablation of these pro-fusion proteins affected calcium signalling activity which subsequently impaired calcineurin activity and Notch signalling (Kasahara et al., 2013). Elevation of Drp1 expression and dephosphorylation of Drp1 at Ser637 also promoted differentiation of C2C12 myoblasts induced by serum starvation. Perturbation of mitochondrial fragmentation by *mdivi-1* or Drp1K38A hinders myogenic differentiation (Kim et al., 2013). Therefore, modulation of mitochondrial morphology may exert an influence on manipulation of differentiation of cardiac stem cells. More recently, expression of prohibitin 2 (PHB2), a pleiotrophic factor mainly localised in mitochondria, mediates homoeostasis and differentiation of ES cells. The level of PHB2 is elevated in undifferentiated mouse ES cells, while the expression was decreased during the differentiation of ES cells. The change in expression pattern of PHBs causes aberrations of mitochondrial functions by modulating the processing of OPA1, yet the effects on OPA1 does not seem to affect differentiation of ES cells (Kowno et al., 2014).

## 6 Limitations and future therapeutic potential of targeting the mitochondrial fusion and fission proteins

The targeting of mitochondrial morphology hails a new cornerstone in designing new therapies for combating cardiovascular disease (see Table 1 for summary). In general the acute inhibition of mitochondrial fission and the acute activation of MFN2 or OPA1 may provide a novel therapeutic strategy in a number of cardiovascular diseases. However, the current potential of modulation mitochondrial morphology as a therapeutic strategy is limited to acute therapeutic manipulation as the chronic modulation of mitochondrial morphology whether that be inhibition of fission or activation of fusion would be deleterious in the long term.

## 7 Summary and conclusions

Mitochondria are now regarded as highly dynamic organelles with multiple roles in cell death and survival. Emerging data suggests that the mitochondrial fusion and fission proteins may provide novel therapeutic targets for treating a variety of cardiovascular diseases including acute IRI, heart failure, left ventricular hypertrophy, and pulmonary arterial hypertension (see Table 1). However, further work is needed to better understand the pleiotropic roles the mitochondrial shaping proteins may play in the cardiovascular system (see Fig. 1), as this may impact on the application of therapeutic strategies which target mitochondrial morphology.

## Funding

S.-B. Ong was funded by a Dorothy Hodgkin Postgraduate Award (Biotechnology and Biological Sciences Research Council) and a Research University Grant (PY/2014/02161) from Universiti Teknologi Malaysia. We thank the British Heart Foundation for their continued support. This work was undertaken at University College London Hospital/University College London, which received a portion of funding from the Department of Health's National Institute of Health Research Biomedical Research Centres funding scheme. This work was also done according to the programme of competitive growth of the Kazan Federal University and the Russian Government.

## Figures and Tables

**Tabel 1:** Potential therapeutic approaches to modulate changes in mitochondrial morphology during pathophysiological cardiac conditions.

**Clinical condition**	**Experimental observation**	**Potential therapeutic approach**
Acute ischaemia/reperfusion (heart, kidney and brain)	Mitochondria undergo fission in response to ischaemia/reperfusion	Inhibit mitochondrial fission using mdiv-1, dynasore, P110 to protect against acute ischaemia/reperfusion injury
Heart failure	MFN1 or MFN2 ablation induces a cardiomyopathy	Activate MFN1, MFN2 or OPA1 to prevent heart failure
Heart failure is associated with reduction in myocardial OPA1 expression
Left ventricular hypertrophy	MFN2 or OPA1 ablation induces left ventricle hypertrophy	Activate MFN2 or OPA1 to prevent left ventricular hypertrophy
Pulmonary arterial hypertension	Vasculoproliferation requires Drp1-mediated fission. Pulmonary arterial hypertension associated with down-regulation of MFN2	Inhibit mitochondrial fission using mdiv-1, dynasore, P110
Activates MFN2
